# Metabolic property of acetaldehyde production from ethanol and glucose by oral *Streptococcus* and *Neisseria*

**DOI:** 10.1038/s41598-019-46790-9

**Published:** 2019-07-18

**Authors:** Ryo Tagaino, Jumpei Washio, Yuki Abiko, Naoko Tanda, Keiichi Sasaki, Nobuhiro Takahashi

**Affiliations:** 1 Division of Oral Ecology and Biochemistry, Tohoku University Graduate School of Dentistry, Sendai, Japan; 2 Division of Advanced Prosthetic Dentistry, Tohoku University Graduate School of Dentistry, Sendai, Japan; 3 Division of Preventive Dentistry, Tohoku University Graduate School of Dentistry, Sendai, Japan

**Keywords:** Pathogens, Oxidoreductases

## Abstract

Acetaldehyde is known to be carcinogenic and produced by oral bacteria. Thus, bacterial acetaldehyde production might contribute to oral cancer. Therefore, we examined bacterial acetaldehyde production from ethanol and glucose under various conditions mimicking the oral cavity and clarified the metabolic pathways responsible for bacterial acetaldehyde production. *Streptococcus mitis*, *S*. *salivarius*, *S*. *mutans*, *Neisseria mucosa* and *N*. *sicca* were used. The bacterial metabolism was conducted at pH 5.0–8.0 under aerobic and anaerobic conditions. The production of acetaldehyde and organic acids was measured with gas chromatography and HPLC, respectively. Bacterial enzymes were also assessed. All of the bacteria except for *S*. *mutans* exhibited their greatest acetaldehyde production from ethanol at neutral to alkaline pH under aerobic conditions. *S*. *mutans* demonstrated the greatest acetaldehyde from glucose under anaerobic conditions, although the level was much lower than that from ethanol. Alcohol dehydrogenase and NADH oxidase were detected in all of the bacteria. This study revealed that oral indigenous bacteria, *Streptococcus* and *Neisseria* can produce acetaldehyde, and that such acetaldehyde production is affected by environmental conditions. It was suggested that alcohol dehydrogenase and NADH oxidase are involved in ethanol-derived acetaldehyde production and that the branched-pathway from pyruvate is involved in glucose-derived acetaldehyde production.

## Introduction

Many epidemiological studies have reported that chronic and heavy alcohol consumption, as well as poor oral hygiene, are strongly correlated with oral cancer^1–4^. However, *in vitro* studies have indicated that ethanol itself is not carcinogenic^5,6^. The mechanism by which poor oral hygiene contributes to the pathogenesis of oral cancer remains unclear. However, a positive association between poor oral hygiene and the occurrence of head and neck cancer was observed in alcohol drinkers^7^, suggesting that alcohol alone is associated with a low risk of cancer, but the co-existence of bacteria and alcohol increases the risk of cancer. When ethanol was added to saliva, greater acetaldehyde production was detected in patients with poor oral hygiene^1^, supporting the assertion that oral bacteria are involved in acetaldehyde production in the oral cavity.

Acetaldehyde is produced from ethanol through the oxidation of ethanol by alcohol dehydrogenase in the liver^8^, and it is widely accepted that the accumulation of acetaldehyde is involved in “hangovers”. Furthermore, acetaldehyde is known to possess carcinogenicity, and the International Agency for Research on Cancer of the World Health Organization classified it as a chemical substance whose carcinogenicity to humans was doubted (group 2B). Many basic studies about the carcinogenicity of acetaldehyde have been performed. In human cells, acetaldehyde can cause sister-chromatid exchanges, gene mutations, and DNA-strand breaks^5,9,10^. In rat hematopoietic stem cells, acetaldehyde damaged chromosomes and caused stem cell mutations^11^. In animal experiments, the oral administration and inhalation of acetaldehyde were suggested to be carcinogenic^12^.

The findings described above suggest that the accumulation of oral bacteria due to poor oral hygiene might increase bacterial acetaldehyde production and subsequently contribute to the development of oral cancer. Recently, oral bacteria, such as *Neisseria*, *Streptococcus*, *Actinomyces*, and *Prevotella* species, and *Candida* species were reported to be able to produce acetaldehyde from ethanol or glucose^13,14^; however, no bacterial species that are specifically associated with oral cancer have ever been found^15–17^. Therefore, other factors might affect the acetaldehyde production of such bacteria in the oral cavity. Environmental factors in the oral cavity, such as the local oxygen concentration, pH, and the types and concentrations of metabolic substrates, are affected by oral hygiene, dietary nutrients, and salivary secretion. Among such environmental factors, the oxygen concentration and pH are known to particularly affect the metabolic activity of oral bacteria^18–20^. Therefore, in order to estimate bacterial pathogenicity, such as the acetaldehyde production of the oral biofilm, it is important to examine not only the bacterial composition of the oral biofilm, but also the metabolic activity of these bacteria while considering the effects of environmental factors on bacterial metabolism^20–22^.

In the present study, we aimed to examine acetaldehyde production by representative examples of the dominant indigenous acetaldehyde-producing bacteria in the oral cavity; i.e., *Streptococcus* and *Neisseria* species, and the effects of oral environmental factors; i.e., pH and the concentrations of oxygen and metabolic substrates, on the metabolic activity of such bacteria. Furthermore, we attempted to suggest the metabolic properties of bacterial acetaldehyde production by detecting the enzymes involved in acetaldehyde production as well as the associated metabolic end-products.

## Results

### Acetaldehyde production from ethanol and the effect of pH and oxygen levels on it

**Figure 1:**
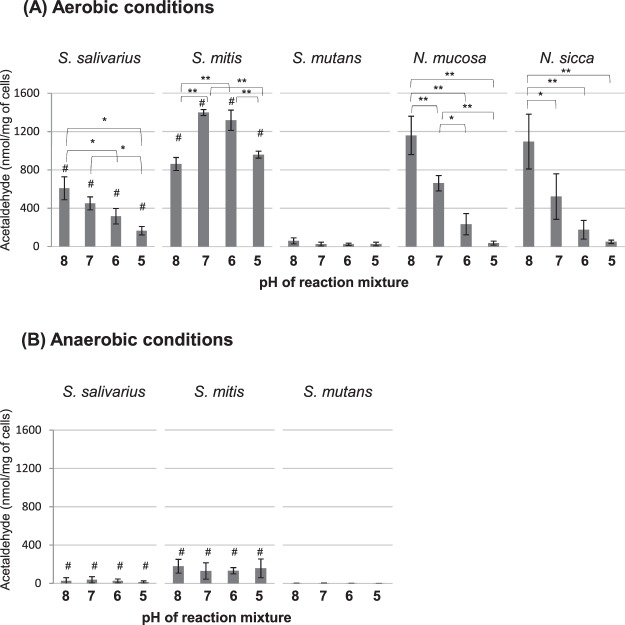
Acetaldehyde production from ethanol for 30 min at 37 °C under aerobic (**A**) or anaerobic (**B**) conditions. Bars, standard deviation; *significant difference (p < 0.05); **significant difference (p < 0.01); ^#^significant difference between aerobic and anaerobic conditions (p < 0.05).

All of the bacterial strains, except for *S*. *mutans*, produced acetaldehyde from ethanol (Fig. 1A,B). *S*. *mitis* and *S*. *salivarius* produced more acetaldehyde under aerobic conditions than under anaerobic conditions (only 4 to 21% of the levels seen during aerobic production). Acetaldehyde production peaked at pH 8.0 in all of the acetaldehyde-producing strains, except for *S*. *mitis*, and it decreased as the pH was lowered (Fig. 1A). The acetaldehyde production of *S*. *mitis* was high at pH 7.0 and 6.0. The effect on pH on acetaldehyde production under anaerobic conditions was unclear (Fig. 1B).

### Acetaldehyde production from glucose and the effect of pH and oxygen levels on it

**Figure 2:**
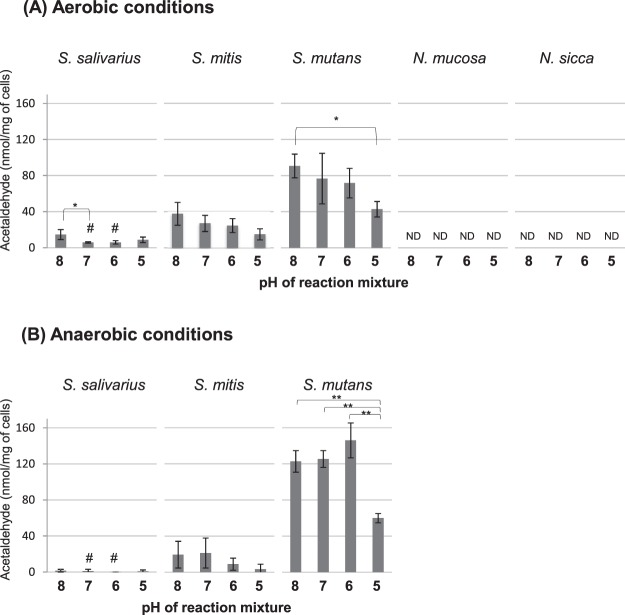
Acetaldehyde production from glucose for 30 min at 37 °C under aerobic (**A**) or anaerobic (**B**) conditions. Bars, standard deviation; ND, below detection limit; *significant difference (p < 0.05); **significant difference (p < 0.01); ^#^significant difference between aerobic and anaerobic conditions (p < 0.05).

The streptococcal strains produced acetaldehyde from glucose and tended to exhibit high activity at pH 8.0, while the *Neisseria* strains did not (Fig. 2A,B). The amounts of acetaldehyde produced from glucose by the streptococcal strains were much lower than those produced from ethanol (1.3 to 5.3%) (Figs 1, 2). Contrary to the findings regarding acetaldehyde production from ethanol, *S*. *mutans* produced more acetaldehyde than the other strains, and greater acetaldehyde production was seen under anaerobic conditions than under aerobic conditions (Fig. 2A,B).

### Alcohol dehydrogenase and NADH oxidase activity

Alcohol dehydrogenase and NADH oxidase activity were detected in all of the strains (Table 1). The *Neisseria* strains exhibited higher alcohol dehydrogenase activity, while the streptococcal strains displayed higher NADH oxidase activity.

**Table 1:** The enzymatic activity of alcohol dehydrogenase and NADH oxidase of *Streptococcus* and *Neisseria* species.

Enzyme	Enzymatic Activity (mU/mg of protein)^a^				
	*S*. *mutans*	*S*. *salivarius*	*S*.* mitis*	*N*. *mucosa*	*N*. *sicca*
Alcohol dehydrogenase	90.2 ± 17.7	9.2 ± 8.7	2.5 ± 0.4	626 ± 384	342 ± 147
NADH oxidase	235 ± 128	198 ± 175	769 ± 690	28.6 ± 17.5	22.8 ± 16.8

^a^Mean ± standard deviation obtained from three independent experiments.

### End-products of ethanol and glucose metabolism by *Streptococcus* strains

**Figure 3:**
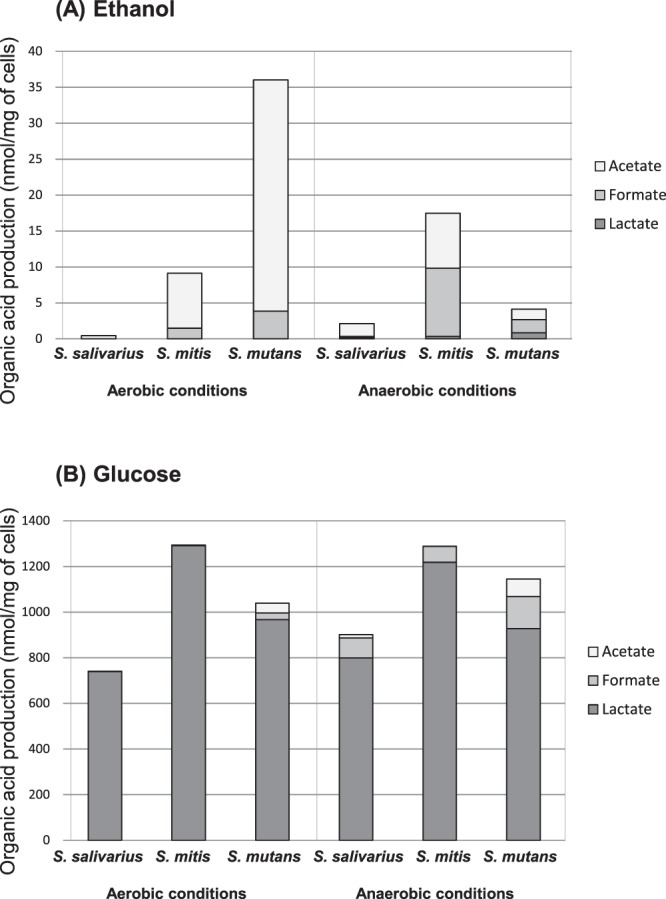
Metabolic end-products produced from ethanol (**A**) or glucose (**B**) by *Streptococcus* species after incubation for 30 min at 37 °C and pH 7.0 under aerobic or anaerobic conditions.

In addition to acetaldehyde, the streptococcal strains produced lactate, acetate, and formate from ethanol as metabolic end-products. *S*. *mutans* produced the highest amounts of end-products under aerobic conditions, with acetate being the main end-product (Fig. 3A). However, these amounts were much smaller than those of acetaldehyde (Fig. 1).

The streptococcal strains mainly produced lactate from glucose, with small amounts of formate and acetate (Fig. 3B). The amounts of these end-products were much higher than the amount of acetaldehyde produced (Fig. 2). *S*. *mutans* produced more formate under anaerobic conditions than the other streptococci.

## Discussion

*S*. *salivarius*, *S*. *mitis*, *N*. *mucosa*, and *N*. *sicca* exhibited greater acetaldehyde production from ethanol under aerobic conditions than under anaerobic conditions (Fig. 1), indicating that aerobic conditions are preferable for acetaldehyde production from ethanol. Only *S*. *salivarius* and *S*. *mitis* produced acetaldehyde from glucose, although the amounts of acetaldehyde produced were much smaller than those produced from ethanol (Fig. 2), confirming that ethanol is the preferred substrate for acetaldehyde production, as reported previously^14^. On the other hand, *S*. *mutans* only produced a small amount of acetaldehyde from ethanol, whereas it produced more acetaldehyde from glucose than the other streptococci (Figs 1, 2). These metabolic properties of *S*. *mutans* will be discussed later. As for the environmental pH, in general bacterial acetaldehyde production was highest at pH 8.0 and decreased as the pH was lowered (Figs 1, 2) (with some exceptions, such as aerobic production from ethanol by *S*. *mitis* and anaerobic production from glucose by *S*. *mutans*).

These results imply that the dominant indigenous oral bacteria, such as non-mutans streptococci and *Neisseria* species^23^, are among those responsible for acetaldehyde production from ethanol in the oral cavity. In addition, the fact that ethanol-derived acetaldehyde production was increased under aerobic conditions and at neutral to weakly alkaline environmental pH suggests that acetaldehyde can be produced in the thin oral biofilm and saliva, even in healthily maintained oral cavities, where oxygen is available enough and pH is neutral to weakly alkaline due to salivary flow. An epidemiological study found a relationship between poor oral hygiene and oral cancer^7,24^, suggesting that thick biofilms might contribute to carcinogenesis. Thick biofilms contain more bacteria than thin biofilms and saliva, and the surfaces of biofilms are exposed to aerobic conditions and are considered to be able to produce acetaldehyde efficiently from ethanol. Therefore, in general poor oral hygiene is a risk factor for acetaldehyde production. In addition, the interior of thick oral biofilms is anaerobic^25^, and thus, acetaldehyde might be produced from glucose by *S*. *mutans* (Fig. 2), which is known to be present in greater numbers in mature and aciduric biofilms^26,27^.

**Figure 4:**
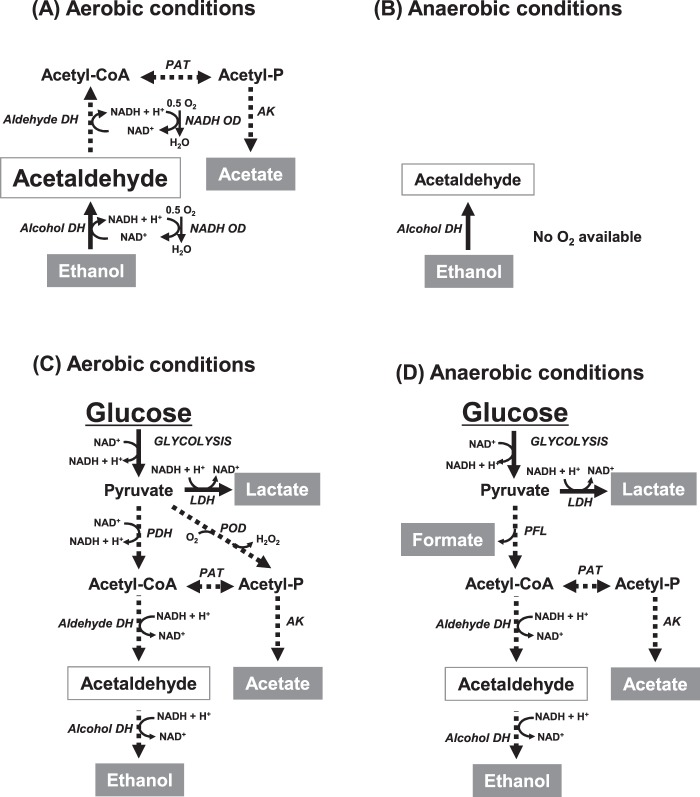
Proposed metabolic pathways related to acetaldehyde production from ethanol under aerobic (**A**) or anaerobic (**B**) conditions or from glucose under aerobic (**C**) or anaerobic (**D**) conditions. Solid arrowed lines, metabolic pathways common for *Streptococcus* and *Neisseria* strains (**A**,**B**) and *Streptococcus* strains (**C**,**D**); Dash arrowed lines, metabolic pathways specific to certain bacteria (see text). LDH, lactate dehydrogenase; PDH, pyruvate dehydrogenase; POD, pyruvate oxidase; PAT, phosphate acetyltransferase; AK, acetate kinase; Aldehyde DH, aldehyde dehydrogenase (acylating); Alcohol DH, alcohol dehydrogenase; NADH OD, NADH oxidase; PFL, pyruvate formate-lyase.

All of the strains examined in this study exhibited alcohol dehydrogenase activity (Table 1), suggesting that bacterial acetaldehyde production from ethanol can be a one-step reaction catalyzed by alcohol dehydrogenase (ethanol + NAD^+^  → acetaldehyde + NADH + H^+^, Fig. 4A,B). In addition, to ensure the efficient continuation of this reaction, the NADH produced seems to be recycled to NAD^+^ by oxidation. All of the strains examined in the current study displayed NADH oxidase activity (Table 1), suggesting that NADH can be oxidized to NAD^+^ by NADH oxidase in the presence of oxygen molecules, and thus, aerobic conditions seem to be necessary for efficient acetaldehyde production from ethanol. Pavlova *et al*.^28^ have reported that alcohol dehydrogenase is responsible to produce acetaldehyde from ethanol in most oral streptococci by using molecular biological methods. Our results support their results.

The optimal pH for alcohol dehydrogenase activity is known to range from 7.5–9.2^29^, which probably explains why greater acetaldehyde production was seen at neutral to weakly alkaline pH (Fig. 1), since the intracellular pH of oral streptococci reflects the environmental pH^30,31^. Acetaldehyde production by *S*. *mitis* was high at pH 7.0 and 6.0, suggesting that the optimal pH for alcohol dehydrogenase activity might differ in this strain, but further studies are needed to examine this.

Despite exhibiting relatively high alcohol dehydrogenase activity, *S*. *mutans* only produced a small amount of acetaldehyde from ethanol (Fig. 1 and Table 1) with trace amounts of acetate and formate (Fig. 3A). The production of acetate suggests that the acetaldehyde produced is further metabolized to acetate via acetyl-CoA and acetyl-phosphate (Fig. 4A) as previously reported^32^, but it seems to be a minor reaction since the amount of acetate was smaller than that of acetaldehyde (Figs 1A, 3A). Over all, these observations suggest that *S*. *mutans* cannot incorporate ethanol efficiently or that ethanol cannot penetrate the cell membrane of *S*. *mutans*, in other words, *S*. *mutans* can be durable against ethanol.

*Streptococcus* strains are known to metabolize glucose to pyruvate through glycolysis, and in the current study they produced various end-products depending on the environment (Fig. 4C,D). Along with acetaldehyde production (Fig. 2), the *Streptococcus* strains mainly produced lactate, with small amounts of acetate and formate (Fig. 3B). Acetaldehyde can be produced via the branched-pathways from pyruvate, which are catalyzed by pyruvate dehydrogenase^33^, pyruvate oxidase^34^, or pyruvate formate-lyase^35^ (Fig. 4C,D). *S*. *mutans*, which produced the highest amounts of formate and acetate from glucose, is suggested to operate these branched-pathways efficiently, and acetaldehyde, a metabolic intermediate of these branched-pathways, might leak out of these bacterial cells, resulting in the observed acetaldehyde production from glucose.

The *Neisseria* strains did not produce acetaldehyde from glucose (Fig. 2A). According to the Kyoto Encyclopedia of Genes and Genomes database, both *N*. *sicca* and *N*. *mucosa* possess gene sequences associated with enzymes related to glycolysis, as well as acid productivity from glucose^36^. However, Muto *et al*.^37^ reported that no aldehyde dehydrogenase activity was detected in these species. Therefore, it is considered that *N*. *sicca* and *N*. *mucosa* can metabolize glucose, but cannot convert it to acetaldehyde.

In conclusion, oral indigenous bacteria, *Streptococcus* and *Neisseria* can produce acetaldehyde, and such acetaldehyde production is affected by environmental conditions. It was suggested that alcohol dehydrogenase and NADH oxidase are involved in ethanol-derived acetaldehyde production and that the branched-pathway from pyruvate is involved in glucose-derived acetaldehyde production.

## Methods

### Bacterial strains

*Streptococcus mitis* (JCM 12971), *Streptococcus salivarius* (JCM 5707), *Neisseria mucosa* (JCM 12292), and *Neisseria sicca* (ATCC 29256) were used in this study. *Streptococcus mutans* (NCTC 10449) was used as a well-studied control.

### Bacterial growth conditions and preparation of the cell suspensions

The *Streptococcus* strains were grown and maintained on blood agar plates (CDC anaerobe 5% sheep blood agar, BD Japan, Japan) or TYG agar plates (agar plates containing 1.7% tryptone, 0.3% yeast extract, 0.5% NaCl, 50 mM potassium phosphate buffer (PPB) [pH 7.0], and 0.5% glucose) and were stored at 4 °C in the air or an anaerobic chamber (80% N_2_, 10% H_2,_ and 10% CO_2_; ANB-18-2E, Hirasawa Works, Japan). Glucose was added through a sterile membrane filter after autoclaving. In the anaerobic growth experiments, the culture media and other reagents were kept under anaerobic conditions for at least 3 days in order to remove any oxygen. The *Streptococcus* strains were cultured in medium at 37 °C, and then 100 μL of the culture were transferred to new medium (40 mL) and further incubated at 37 °C until the logarithmic growth phase. The bacterial cells were harvested via centrifugation (10,000 rpm for 7 min at 4 °C) and washed 3 times with washing buffer (2 mM PPB [pH 7.0] containing 75 mM KCl, 5 mM MgCl_2_, and 75 mM NaCl), before being suspended in the same buffer. The bacterial cell concentrations of the suspensions were adjusted on the basis of their optical density (OD) at a wavelength of 660 nm (to an OD of 5.0). The anaerobically cultured cells remained in the anaerobic chamber throughout the culture procedure, while the aerobically cultured cells were prepared in the air throughout the culture procedure.

The *Neisseria* strains were grown and maintained on blood agar plates (Columbia 5% sheep blood agar, BD Japan, Japan) and stored at 4 °C in the air. They were cultured on blood agar plates at 37 °C, transferred to new blood agar plates, and then further incubated at 37 °C for 12 hours in the air. The bacterial cells were harvested via centrifugation (10,000 rpm for 7 min at 4 °C) and washed 3 times with washing buffer (2 mM PPB [pH 7.0] containing 75 mM KCl, 5 mM MgCl_2_, and 75 mM NaCl), before being suspended in the same buffer. The cell suspensions were adjusted to OD of 7.5 for *N*. *mucosa* and 6.2 for *N*. *sicca*, as described above. The aerobically cultured cells were prepared in the air throughout the culture procedure.

### Measurement of bacterial acetaldehyde production

The reaction mixture used to assess bacterial acetaldehyde production contained 850 μL of the bacterial cell suspension, 100 μL of substrate (100 mM glucose or 11 mM ethanol), and 50 μL of PPB (pH: 8.0, 7.0, 6.0, or 5.0). Each reaction mixture was collected in a tube with a silicone cap, and the tube was completely closed off. The reaction was started via the addition of the metabolic substrate and incubated at 37 °C for 30 min. The reaction was stopped via the injection of 100 μL of 7 M phosphoric acid using a sterile needle (Terumo injection needle 27 G × 3/4, Terumo Corporation, Japan) and syringe (1 mL Terumo syringe for tuberculin, Terumo Corporation, Japan) through the silicone cap, and the mixture was shaken vigorously. The headspace gas of the tube was collected using a gas-tight syringe (10 ml NORM-JECT, HENKE- SASS WOLF, Tuttlingen, Germany) and was diluted with nitrogen gas. Then, the concentrations of acetaldehyde were measured using a sensor gas chromatograph (SGEA-P2, FIS Inc., Japan). The reaction mixture was stored at 4 °C until it was used to measure the levels of organic acids, as described below. The aerobic production of acetaldehyde in air was measured using aerobically cultured cells, while the anaerobic production of this molecules was measured in an anaerobic chamber (80% N_2_ and 10% H_2_; ANB-180R, Hirasawa Works, Japan) using anaerobically cultured cells.

### Measurement of bacterial alcohol dehydrogenase and NADH oxidase activity

The aerobically cultured bacteria were harvested as described above and stored as pellets at −30 °C until they were used. Each pellet was thawed and suspended in a buffer containing 75 mM KCl, 75 mM NaCl, 2 mM MgCl_2_, and 2 mM PPB (pH 7.0), before being oscillated with an ultrasonic disruption apparatus (Insonator 201 M, KUBOTA CORPORATION, Japan) (2 A, 190 W, at 4 °C for 7 min) and centrifuged to remove any cell membranes or intact cells. The prepared cell-free extracts were used to measure enzymatic activity. NAD^+^-dependent alcohol dehydrogenase activity was assessed using an assay kit (alcohol dehydrogenase activity assay kit, K787-100, Funakoshi, Tokyo, Japan), according to the manufacturer’s instructions. The enzymatic reactions were performed in an anaerobic chamber (80% N_2_ and 10% H_2_; ANB-180R) in order to avoid masking by NADH oxidase activity.

NADH oxidase activity was measured according to the method of Reusch and Burger^38^, with minor modifications. In a quartz cell, 870 μL of 50 mM PPB (pH 7.0) containing 2 mM NADH was prepared, and the reaction was started via the addition of 30 μL of the cell-free extract. The activity was monitored with a spectrophotometer (UV SPECTROPHOTOMETER UV-1800, Shimadzu Co. Ltd., Japan) at 340 nm and 30 °C in the air.

The protein concentrations of the cell-free extracts were measured using an assay kit (Takara BCA protein assay kit T9300, Takara Bio Inc., Japan), according to the manufacturer’s instructions.

### Measurement of bacterial organic acid production

The reaction mixtures used to assess acetaldehyde production from ethanol or glucose at pH 7.0 were centrifuged at 10,000 rpm at 4 °C for 7 min, and the supernatants were collected. The organic acid (acetate, lactate, and formate) levels of the samples were then measured as described previously^39,40^. After the bacteria were removed using a filter (pore size: 0.20 µm; polypropylene; Toyo Roshi Ltd., Japan), the organic acid levels of the samples were determined using high-performance liquid chromatography (Shimadzu Prominence LC-20AD, Shimadzu Co. Ltd., Japan).

### Statistical analysis

In the statistical analyses, the significance of the differences among multiple groups were analyzed using Tukey’s test, whereas the significance of the differences between pairs of groups were analyzed using the paired t-test, and *p* values of <0.05 were considered statistically significant (StatFlex Ver. 6 and Microsoft^®^ Excel^®^ Ver. 14).
